# Special Issue “Liver Fibrosis: Molecular Pathogenesis, Diagnosis and Treatment”

**DOI:** 10.3390/ijms27177922

**Published:** 2026-09-05

**Authors:** Manuel Morales-Ruiz

**Affiliations:** 1 Biochemistry and Molecular Genetics Department, Hospital Clínic of Barcelona, Fundació de Recerca Clínic Barcelona- Institut d’Investigacions Biomèdiques August Pi i Sunyer (IDIBAPS), Centro de Investigación Biomédica en Red de Enfermedades Hepáticas y Digestivas (CIBERehd), 08036 Barcelona, Spain; morales@clinic.cat; 2 Biomedicine Department, Faculty of Medicine and Health Sciences, University of Barcelona, 08036 Barcelona, Spain

## 1. The Clinical and Molecular Challenge of Hepatic Fibrogenesis

Chronic liver disease (CLD) represents an escalating global health crisis, with liver disease causing approximately 2 million deaths annually worldwide; notably, metabolic dysfunction-associated fatty liver disease affects roughly 30% of the global population [1,2]. Regardless of the underlying etiology, whether driven by metabolic dysfunction, viral hepatitis, autoimmune conditions, or alcohol abuse, the progression of CLD is characterized by hepatic fibrogenesis. This dynamic pathophysiological process entails the continuous activation of hepatic stellate cells (HSCs), the recruitment of innate and adaptive immune cells, and the subsequent excessive deposition of extracellular matrix (ECM) proteins, leading to severe architectural distortion and, ultimately, end-stage cirrhosis, portal hypertension, and hepatocellular carcinoma (HCC) [3,4,5,6].

Despite major advances in understanding the cellular and molecular mechanisms of liver injury, no effective targeted anti-fibrotic treatments exist at present [7]. The translation of preclinical findings to clinical settings has been hampered by the spatial, metabolic, and epigenetic heterogeneity of fibrotic livers, which is poorly reproduced by conventional two-dimensional cultures and only partially captured by standard animal models.

**Figure 1:**
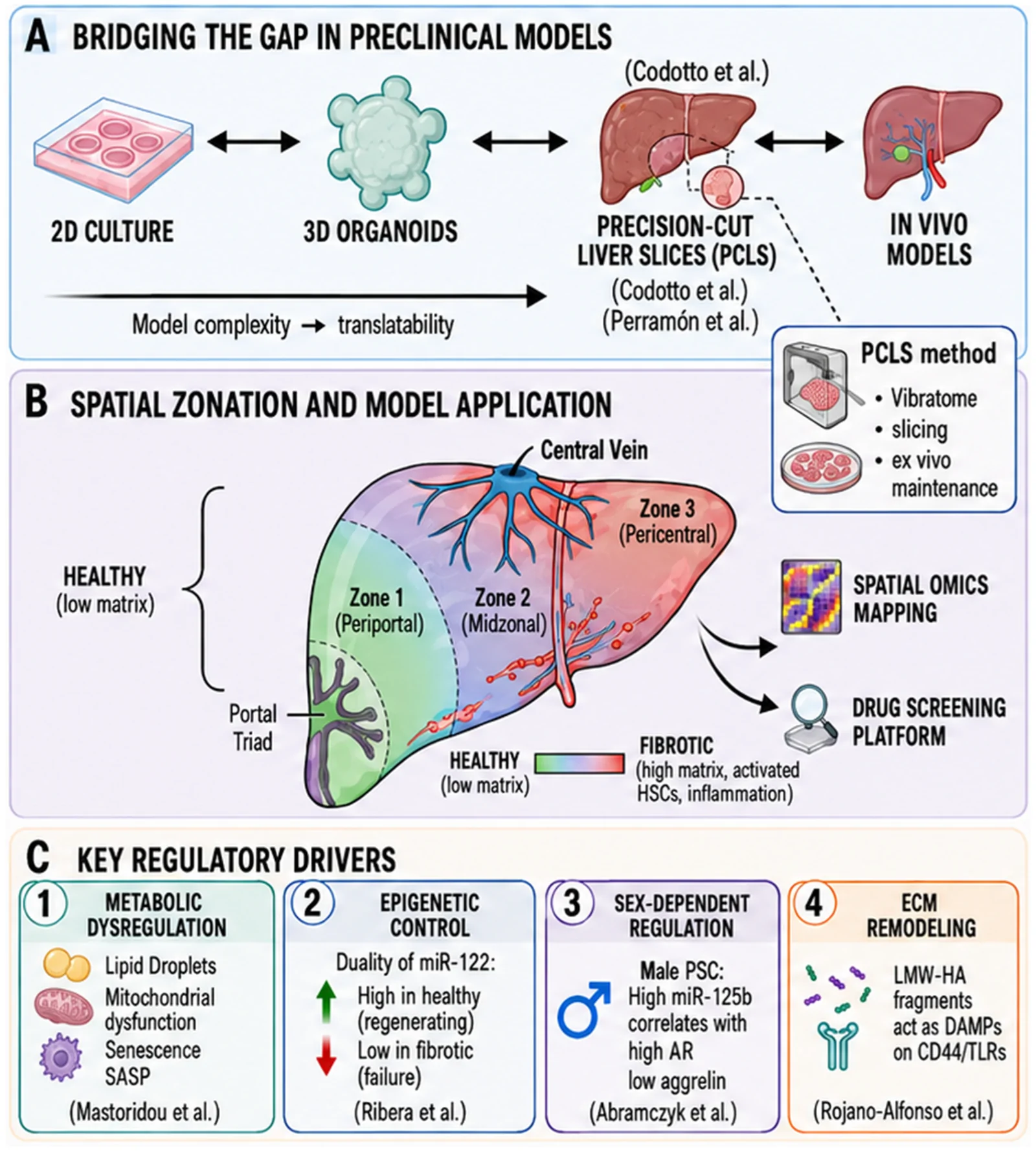
Integrated preclinical models and spatial complexity of liver fibrosis. (**A**) Increasing model complexity and translational relevance from two-dimensional cultures and three-dimensional organoids to precision-cut liver slices (PCLS) and in vivo models. The inset summarizes PCLS preparation and ex vivo maintenance. (**B**) Hepatic zonation from the periportal to the pericentral region and its relationship with the transition from healthy, low-matrix tissue to fibrotic areas characterized by extracellular matrix accumulation, HSC activation, and inflammation. Spatial omics and drug-screening platforms enable region-specific characterization and therapeutic testing. (**C**) Regulatory mechanisms highlighted in this Special Issue, including metabolic dysfunction and senescence, miR-122-mediated epigenetic regulation, sex-dependent miR-125b/androgen receptor signaling, and extracellular matrix remodeling by low-molecular-weight hyaluronic acid fragments.

This Special Issue of the International Journal of Molecular Sciences aims at addressing these considerable gaps. Bringing together ten cutting-edge, multidisciplinary studies, this collection covers advanced spatial multi-omics, innovative ex vivo human and rodent models, non-invasive viscoelastic diagnostics, and the elucidation of novel epigenetic and metabolic therapeutic targets (Figure 1), providing a valuable contribution to both our current understanding and the immediate future of precision hepatology and translational anti-fibrotic research.

## 2. Decoding the Fibrotic Niche: Advanced Modeling and Spatial Multi-Omics

Developing effective therapies for liver fibrosis requires models that accurately mirror the liver’s structural architecture and cellular diversity. Codotto et al. [Contribution 1] point out how the establishment of robust single-cell RNA sequencing (scRNA-seq), single-nucleus RNA sequencing (snRNA-seq), and spatial transcriptomics (ST) has fundamentally transformed the mapping of regional heterogeneity within the fibrotic niche, allowing researchers to accurately distinguish between mesenchymal cell sub-populations (e.g., portal vein-associated HSCs versus central vein-associated HSCs) and understand their specific topographies and their differential roles in producing collagen. The authors also emphasize the imperative transition from standard 2D cultures to sophisticated 3D models, such as induced pluripotent stem cell (iPSC)-derived hepatobiliary organoids and precision-cut liver slices (PCLS). These 3D systems robustly replicate human pathophysiology, including lipid accumulation, ballooning, and inflammation, while preserving the spatial functional zonation critical for evaluating pharmacological interventions.

Bringing this advanced modeling concept to the laboratory bench, Perramón et al. [Contribution 2] provide a rigorous validation of PCLS as a translational preclinical platform beyond 3D models. They examine vibratome-generated, 250 μm thick PCLS from healthy and cirrhotic rats (treated with carbon tetrachloride) and human biopsies, showing that when maintained under stringently optimized and controlled ex vivo conditions (40% oxygen tension), the fibrotic slides retained their respective pathogenic gene signatures for more than 48–72 h. Importantly, increased transcriptomic expressions of *Col1a2*, *Col3a1*, *αSMA*, and *Mmp2*, the key ECM remodeling markers, were evident in the fibrotic slides. The translational utility of this model was further corroborated by demonstrating its robust responsiveness to pro-inflammatory external stimuli, such as lipopolysaccharides (LPS), which induced significant overexpression of *Nos2* and *Il1β*. More strikingly, the authors proved the possibility of adenoviral delivery of dominant negative sPDGFRβ in the PCLS, efficiently inhibiting fibrotic signaling and opening avenues of high-throughput ex vivo studies for fibrotic drug screening.

## 3. Systemic Interplay: Viscoelasticity, Trace-Element Metabolism, and Non-Invasive Diagnostics

The early and accurate clinical staging of fibrosis is paramount for patient management. Shear-wave elastography provides the gold standard for measuring liver stiffness in vivo, but is heavily restricted in morbidly obese patients, as the wall thickness hinders wave propagation. Biasin et al. [Contribution 3] published a pilot study that tried to address this problem by combining rheology with Low-Field Nuclear Magnetic Resonance (LF-NMR). They studied liver biopsies taken from patients with metabolic and bariatric surgery, measuring both the elastic properties, such as the shear modulus *G* and the critical stress τ_c_, and the complex viscoelastic properties via (G_a_*). Their methodology established that pathological samples exhibit markedly elevated *G*_a_*, *G*, and *τ*_c_ values, which closely aligned with the transcriptomic upregulation of profibrotic markers such as *Col1a1*, Fibronectin (*FN*), *MMP2*, *MMP9*, and *E2F1*. Concurrently, LF-NMR measurements revealed that reduced spin–spin relaxation time (*T*_2m_) strongly correlated with iron accumulation, confirmed biochemically by elevated Ferritin (*FTH1*) and Hepcidin (*HEPC*) expression. By this sensitive biophysical and simultaneous combination of methods, they demonstrated the inverse correlation of *T*_2m_ with increases in viscoelastic moduli (Ga*/*G*) and stiffness, with pathological iron accumulation being a decisive factor in the progression of metabolic dysfunction-associated steatotic liver disease (MASLD).

At the diametrically opposed side, in patients with end-stage liver failure (ESLF), metabolic exhaustion is exemplified by a study from Adamus and colleagues [Contribution 4] that investigated the homeostasis of molybdenum (Mo), an essential trace element along with that of the key molybdenum-containing enzymes Xanthine Oxidase (XO) and Aldehyde Oxidase (AO). Molybdenum, incorporated into the highly conserved molybdenum cofactor (MoCo), is fundamental for purine catabolism, xenobiotic detoxification, and the maintenance of redox homeostasis. Analyzing a cohort of liver transplant recipients stratified by etiology (ALD, HBV/HCV, PSC, WD, and PBC), the researchers described a significant, uniform depletion in total hepatic molybdenum content and a concurrent significant drop in XO activity when compared to healthy liver donors. Interestingly, while both parameters decreased, the absence of a direct linear correlation between Mo content and XO activity in the ESLF cohort suggests a profound uncoupling of trace-element availability from functional MoCo biosynthesis and enzyme maturation. This demonstrates that the architectural remodeling, altered membrane transport, and profound hepatocyte loss inherent to advanced cirrhosis fundamentally incapacitate the fundamental liver activities aimed at maintaining trace-element and redox homeostasis.

## 4. The Cellular Nexus: Senescence, Lipid Metabolism, and Epigenetic Heterogeneity

A recurring theme in this Special Issue is the interaction between chronic metabolic stress, cellular aging, and epigenetic dysregulation. Mastoridou et al. [Contribution 5] review the bidirectional relationship between cellular senescence and lipid droplet dynamics in MASLD. Early lipid overload may trigger an adaptive PPARα-dependent increase in fatty acid oxidation, but sustained lipotoxicity promotes stress-induced premature senescence, mitochondrial dysfunction, reactive oxygen species generation, and *p16INK4a*- and *p53*/*p21*-mediated cell-cycle arrest. Senescent hepatocytes also upregulate CD36, increasing lipid uptake, and develop a senescence-associated secretory phenotype driven mostly by NF-κB. The resulting release of inflammatory cytokines and metalloproteinases amplifies local injury and spreads senescence to neighboring cells, promoting progression from steatosis to metabolic dysfunction-associated steatohepatitis (MASH).

Ribera et al. [Contribution 6] investigated the context-dependent role of miR-122, the most abundant hepatic microRNA, using a CRISPR/Cas9 germline knockout mouse model. After two-thirds partial hepatectomy in healthy liver, the loss of miR-122 accelerated regeneration through increased Cyclin D1 and RhoA expression and more efficient cytokinesis. After undergoing chronic CCl_4_-induced injury, however, mice became susceptible to developing severe fibrosis and cirrhosis-like remodeling. Following hepatectomy, these mice retained proliferative signaling but developed bioenergetic failure, loss of hepatocyte function, and high mortality, a state confirmed with proteomic studies that showed the involvement of mitochondrial dysfunction, redox imbalance, and inflammation, indicating the severity of the disease. Thus, miR-122 inhibits proliferation in a healthy liver and is essential to metabolic viability and effective repair during chronic injury.

Abramczyk et al. [Contribution 7] investigated sex-dependent regulation in primary sclerosing cholangitis (PSC), focusing on miR-125b, androgen receptor (AR) signaling, and fibrogenic pathways. Male patients, particularly those with advanced fibrosis, showed increased miR-125b in serum and liver tissue. Its expression correlated positively with AR and inversely with the protective peptide apelin and transforming growth factor-beta. Overexpression of miR-125b in hepatocytes and cholangiocytes caused increased AR mRNA and lower p53 and apelin mRNA. Ursodeoxycholic acid treatment lowered serum miR-125b levels over three years, suggesting that part of its effect may involve the restoration of this epigenetic balance. These findings identify miR-125b as a potential mediator of male-predominant disease severity in PSC.

ACE, angiotensin-converting enzyme; AR, androgen receptor; DAMPs, damage-associated molecular patterns; ECM, extracellular matrix; HA, hyaluronic acid; HAS2, hyaluronan synthase 2; HMW-HA, high-molecular-weight hyaluronic acid; HSC, hepatic stellate cell; LMW-HA, low-molecular-weight hyaluronic acid; PCLS, precision-cut liver slices; PSC, primary sclerosing cholangitis; RAAS, renin–angiotensin–aldosterone system; SASP, senescence-associated secretory phenotype; TLRs, Toll-like receptors; 4-MU, 4-methylumbelliferone.

## 5. Innovative Therapeutic Interventions and Pharmacological Targets

**Figure 2:**
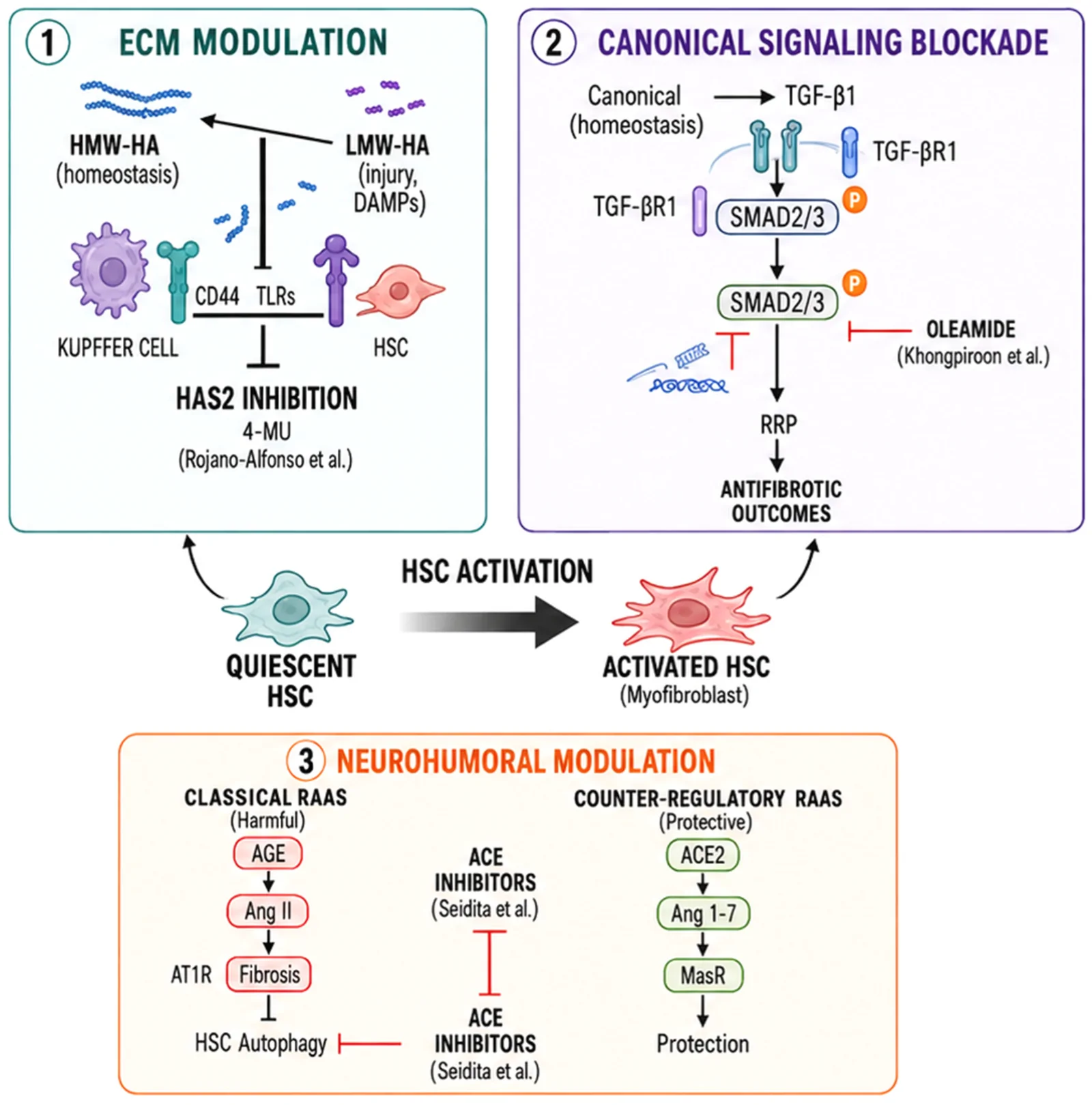
Mechanistic therapeutic strategies for halting liver fibrogenesis. Hepatic stellate cell (HSC) activation and transition from a quiescent phenotype to an activated myofibroblast-like state can be targeted through complementary therapeutic approaches. ① Extracellular matrix modulation: tissue injury promotes the generation of low-molecular-weight hyaluronic acid fragments, which act as damage-associated molecular patterns through CD44 and Toll-like receptors in Kupffer cells and HSCs. Inhibition of hyaluronan synthase 2 by 4-methylumbelliferone may attenuate this profibrogenic response. ② Canonical signaling blockade: inhibition of the TGF-β1/TGF-β receptor/SMAD2/3 pathway, including the effects of oleamide, reduces downstream profibrotic transcriptional responses and promotes anti-fibrotic outcomes. ③ Neurohumoral modulation: suppression of the classical renin–angiotensin–aldosterone system axis, including angiotensin II/AT1 receptor signaling, together with enhancement of the counter-regulatory ACE2/angiotensin-(1–7)/Mas receptor axis, may reduce fibrosis, modulate HSC autophagy, and promote hepatoprotection.

Rojano-Alfonso et al. [Contribution 8] review the dynamic role of hyaluronic acid (HA) in the hepatic sinusoidal niche. Beyond its structural function in the extracellular matrix, HA acts as a size-dependent immune modulator. High-molecular-weight HA, produced mainly by hyaluronan synthase 2, supports homeostasis. However, upon tissue damage, HA fragments of lower molecular weight are synthesized which, in turn, act as damage-associated molecular patterns. Signaling via CD44 and Toll-like receptor, these fragments activate NF-κB, stimulate the production of inflammatory cytokines, and induce the transdifferentiation of HSCs (Figure 2). HA additionally promotes the polarization of neighboring macrophages to a phenotype associated with hepatocellular carcinoma. As such, the pharmacological inhibition of HA synthesis with 4-methylumbelliferone could effectively block pathogenic-matrix–immune crosstalk.

The vast therapeutic potential of repurposing existing, clinically approved medications is examined by Seidita et al. In their comprehensive review [Contribution 9], they investigate the therapeutic efficacy of Renin–Angiotensin–Aldosterone System (RAAS) inhibitors, specifically Angiotensin-Converting Enzyme Inhibitors (ACEIs) and Angiotensin Receptor Blockers (ARBs), in the attenuation of liver fibrosis within the context of MASLD. The authors refer to the overwhelming supporting data from retrospective studies which suggest that ACEI usage confers lower risk to patients, especially those with concurrent chronic kidney disease, of developing liver-related events and HCC. Mechanistically, the classical RAAS pathway (generating Angiotensin II via ACE) promotes vasoconstriction, intense inflammation, and fibrosis through the AT1 receptor. Crucially, Angiotensin II signaling directly enhances the autophagy-dependent activation of HSCs, providing the necessary metabolic fuel (via lipid droplet degradation and beta-oxidation) for myofibroblast transdifferentiation and ECM secretion. By blocking this axis, ACEIs elevate ACE2 expression, shunting the pathway toward the production of Angiotensin 1–7, which interacts with the MAS receptor to promote vasodilation and anti-fibrotic responses (Figure 2). Hence, RAAS blockade is reachable and appears to be a reasonable strategy of stopping autophagy in HSCs, so the progression of fibrosis is interrupted.

Khongpiroon et al. [Contribution 10] identified oleamide (OLA), a naturally occurring amide isolated from the leaves of *Moringa oleifera* Lam., as a potential anti-fibrotic compound. In TGF-β1-activated LX-2 hepatic stellate cells, OLA suppressed activation with limited cytotoxicity, and this result is strongly supported by molecular docking prediction of favorable interactions with TGF-β receptors and SMAD proteins. In this context, RT-PCR and Western blotting experiments confirmed reduced SMAD2/3 phosphorylation, downregulation of major profibrotic genes, decreased IL-6 and IL-8 secretion, and gelatinase activity (Figure 2). These complementary in vitro and in silico findings support oleamide as a promising plant-derived candidate, although in vivo validation is still required.

## 6. Future Directions

Several critical priorities emerge from this Special Issue for advancing the field.

First, longitudinal studies combining senescence markers, metabolic flux analyses, and proteomic profiling in experimental MASLD models are essential for temporally mapping the transition from steatosis to MASH to cirrhosis and understanding at what stages interventions remain effective. Mechanistic and molecular insights need to be coupled with careful clinical validation in prospectively enrolled cohorts of patients stratified by distinct disease stages and different genetic background.

Second, the integration of omics data with physiologically realistic tissue models requires systematic development. Future studies should combine scRNA-seq and spatial transcriptomics datasets with patient-derived PCLS or organoid systems to validate transcriptomic findings functionally and test therapeutic candidates in genetically relevant contexts. This integrated approach would reconcile the bridges between basic science and translational research.

Third, development of biomarker-driven patient stratification strategies should accelerate clinical trial design. Given the stage-dependent efficacy of senolytic agents, fibrosis-modifier gene signatures, and sex-dependent signaling differences documented in this Special Issue, future trials must incorporate baseline stratification by disease stage, sex, and genetic/epigenetic markers. This will improve the chances of capturing potential efficacy and permit more efficient resource utilization.

Fourth, research into combination therapeutic strategies should be prioritized. Senescence, lipid metabolism, inflammation, and fibrotic signaling are so closely linked that blocking single pathways may likely be insufficient. Rational combinations of senescence-targeting agents, metabolic modulators, and anti-inflammatory therapies should be rationally designed and tested preclinically and clinically to evaluate the benefits of this holistic strategy.

Finally, investigation of rare liver diseases and underrepresented populations in fibrosis research must be expanded. The unique biochemical and immunological alterations in PSC, primary biliary cholangitis, and Wilson’s disease highlighted in this Special Issue offer opportunities to uncover disease-specific pathogenic mechanisms with broad applicability. Similarly, sex- and ethnicity-dependent differences in fibrosis progression require dedicated investigation to ensure equitable translation of therapeutic advances.

## 7. Conclusions

Taken together, the papers in this Special Issue show a field moving toward mechanism-guided precision hepatology. The most important shift is conceptual: liver fibrosis is better understood as a dynamic tissue ecosystem shaped by metabolism, matrix biology, inflammation, regeneration, and context-dependent heterogeneity than as a single pathway disorder. The most urgent task now is translational: to convert that mechanistic richness into stratified, reproducible interventions that improve liver function and clinical outcomes, not only surrogate markers.
